# Cognitive decline monitoring through a web-based application

**DOI:** 10.3389/fnagi.2023.1212496

**Published:** 2023-10-05

**Authors:** Laura V. Sánchez-Vincitore, Daniel Cubilla-Bonnetier, Hugo Marte-Santana, Jon Andoni Duñabeitia

**Affiliations:** ^1^Laboratorio de Neurocognición y Psicofisiología, Universidad Iberoamericana (Unibe), Santo Domingo, Dominican Republic; ^2^Centro de Investigación Nebrija en Cognición, Universidad Nebrija, Madrid, Spain; ^3^Department of Language and Culture, The Arctic University of Norway, Tromsø, Norway

**Keywords:** age-associated cognitive decline, cognitive decline, aging, working memory, physical activity, sleep quality, naturalistic environment

## Abstract

Cognitive decline usually begins after individuals reach maturity, which is more evident in late adulthood. Rapid and constant cognitive screenings allow early detection of cognitive decline and motivate individuals to participate in prevention interventions. Due to accelerated technological advances, cognitive screening and training are now available to the layperson using electronic devices connected to the internet. Large datasets generated by these platforms provide a unique opportunity to explore cognitive development throughout life and across multiple naturalistic environments. However, such data collection mechanisms must be validated. This study aimed to determine whether the data gathered by commercial visuospatial and phonological working memory tests (CogniFit Inc., San Francisco, USA) confirm the well-established argument that age predicts cognitive decline. Data from 3,212 participants (2,238 females) who were 45 years old or older were analyzed. A linear regression analysis explored the relationship between age and working memory while controlling for gender, sleep quality, and physical activity (variables that are known to affect working memory). We found that age negatively predicts working memory. Furthermore, there was an interaction between age and gender for visuospatial working memory, indicating that although male participants significantly outperformed females, the relationship between age and working memory differs for females and males. Our results suggest that the computerized assessment of visuospatial and phonological working memory is sensible enough to predict cognitive functions in aging. Suggestions for improving the sensitivity of self-reports are discussed. Further studies must explore the nature of gender effects on cognitive aging.

## 1. Introduction

Age-associated cognitive decline refers to the non-pathological deterioration of cognitive processes through aging, usually related to fluid intelligence –such as memory, processing speed, and reasoning– but not necessarily crystallized abilities –like phonological and numerical abilities or general knowledge (Deary et al., 2009; Murman, 2015, but see Buades-Sitjar et al., 2022). Most people present a relatively slow deterioration that is detectable at the decade timescale, and those whose cognitive abilities deteriorate faster are more likely to have a type of neuropathology (Hayden et al., 2011). In addition, research has found that cognitive function is associated with health-related quality of life in older people (Kazazi et al., 2018). In the preservation of these functions, there may also be mediating factors such as those underlying cognitive reserve (Adam et al., 2013; Guzmán-Vélez and Tranel, 2015; Alvares Pereira et al., 2022). Therefore, cognitive functioning has become a priority for interventions that target the wellbeing of elderly individuals.

Among the cognitive abilities that undergo decline with age, working memory has gained considerable attention in recent years. Working memory refers to the multi-component cognitive system that allows individuals to temporarily hold and manipulate information necessary to perform complex cognitive tasks (Baddeley, 2017). This multi-component system consists of a phonological loop –that stores and rehearses verbal information–a visuospatial sketchpad –that stores and manipulates visual and spatial information and a central executive –that coordinates both. Working memory is crucial in everyday tasks such as language, problem-solving, and decision-making. Studies have shown an inverse relationship between age and working memory, demonstrating a decline in working memory with increasing age (Bopp and Verhaeghen, 2005, 2020; Pliatsikas et al., 2019). Furthermore, Pliatsikas et al. observed an interaction between age and gender, with men exhibiting a more pronounced decline in working memory than women as a function of age. Finally, evidence suggests that phonological working memory resists aging, while age affects visuospatial working memory more (Kumar and Priyadarshi, 2013).

According to the International Psychogeriatric Association, the inclusion criteria for age-associated cognitive decline are (1) self-perception of cognitive decline for at least 6 months and (2) low scores at least one standard deviation below age and education in cognitive tests. When using these criteria, the prevalence of age-related cognitive decline is greater than 20% in people older than 64 and increases linearly with age (Hänninen et al., 1996; Busse et al., 2003; Schönknecht et al., 2005). In addition, conversion rates from age-associated cognitive decline to dementia –a pathological form of cognitive decline– are around 30%, as reported in various longitudinal studies (Ritchie et al., 2001; Busse et al., 2003).

In addition to aging, sociodemographic, health, and lifestyle factors are associated with cognitive decline. For example, considering sociodemographic factors, some studies have reported that low socioeconomic positions, low educational attainment (Deary et al., 2009), and neighborhood deprivation are associated with cognitive decline (Clarke et al., 2015). Regarding health factors, research has shown that the most common factors associated with cognitive decline were metabolic syndrome (Frisardi, 2014), vascular risk (Ganguli et al., 2014), hypertension (Naing and Teo, 2020), diabetes (Feinkohl et al., 2015), obesity (Nguyen et al., 2014; Dye et al., 2017), inflammation (Cunningham and Hennessy, 2015), and oxidative stress (Baierle et al., 2015). Opposingly, the lifestyle factors that have been shown to prevent cognitive decline are regular physical activity (Angevaren et al., 2010; Lee et al., 2015; Song and Park, 2022), nutrition (Dauncey, 2014; Smith and Blumenthal, 2016), sleep quality (Brocklebank et al., 2021; Joo et al., 2021), and participation in leisure activities (Wang et al., 2013; Yates et al., 2016).

Being able to track age-associated cognitive decline could help identify those individuals at risk for dementia. Identifying them at the pre-clinical stage is a priority for health professionals, given that early detection allows clinicians to design better interventions and prevention strategies (Cruz-Oliver and Morley, 2010). However, there are multiple challenges associated with such identification. One of these is that cognitive functions usually change gradually and continuously across the lifespan. Severity might be among the few differentiating factors between non-pathological age-related cognitive decline and early onset of dementia (Murman, 2015). Salthouse (2019) indicates that the timing of cognitive decline onset is critical to determine the best course of action. Describing typical cognitive aging, including age-associated cognitive decline, is crucial to detect such onset. To understand the heterogeneity of cognitive aging across individuals, Tucker-Drob (2019) recommends framing it as a continuous model that involves analyzing changes in cognitive trajectories over time. Even though gold-standard diagnostic tools for dementia (like the Mini-Mental State Test) are essential, relevant, and valid, a continuous model that constantly addresses cognitive functioning could detect changes in earlier stages, providing opportunities for prevention and early interventions (Grodstein, 2012).

Continuous monitoring of cognitive functions can be achieved through computerized assessments (see Asensio and Duñabeitia, 2023), which enable the collection of vast amounts of data from a single patient, overcoming the impracticalities related to having multiple face-to-face interviews. Allard et al. (2014) suggest that measuring cognition over time on mobile devices effectively complements traditional testing. Given the latest advances in artificial intelligence, massive amounts of data can be quickly processed and analyzed, contributing to dementia diagnosis and treatment (Khodabandehloo et al., 2021; Revathi et al., 2022). CogniFit (CogniFit Inc., San Francisco, USA) is a digital health platform offering computerized cognitive assessments and interventions for users, researchers, and practitioners. Assessments and interventions are available for desktop and laptop computers and mobile devices like tablets and smartphones. CogniFit’s general cognitive assessment (CAB) is an online neurocognitive assessment that aims to evaluate cognitive functions in the following domains: attention, perception, memory, executive functions, coordination, and physical, psychological, and social wellbeing. This instrument has been widely used for cognitive assessment (Yaneva and Mateva, 2017; Sánchez-Sansegundo et al., 2021; Berbegal et al., 2022; Tapia et al., 2022, 2023a,2023b; Duñabeitia et al., 2023).

Given the availability of large databases from CogniFit users, with diverse naturalistic environments, as opposed to controlled research environments, in the current study, we investigated the validity of user-generated data to capture cognitive decline across the lifespan. Furthermore, with the technology available for continuous monitoring of cognitive functions across different ages, this study aimed to determine whether the data gathered by commercial visuospatial and phonological working memory tests confirm the well-established argument that age predicts cognitive decline.

## 2. Materials and methods

### 2.1. Participants

The dataset for this analysis was extracted from a more extensive CogniFit user database of 27,619 users. To ensure the dataset was appropriate for analysis, we excluded participants who were younger than 45 years of age. This resulted in a final sample of 3,212 participants (2,238 women; mean age = 54.99; SD = ±8.49). No further exclusion criteria were applied as the study aimed to explore user-generated data.

Most of the participants spoke English (55.35%), followed by Spanish (16.73%), Russian (9.87%), French (7.72%), German (2.52%), and other languages (7.81%). In addition, most participants lived in Europe (52.71%), followed by North America (29.47%) and Latin America (8.61%).

### 2.2. Instruments

#### 2.2.1. Visuospatial working memory

Participants completed the CogniFit game “Glowing Circles” to measure this ability. This task is a variant of the well-known Corsi test, a widely used neuropsychological assessment tool for evaluating visuospatial working memory (Kessels et al., 2000). In the Corsi test, an experimenter taps a series of squares on a board, and participants must reproduce the same tapping sequence. Similarly, in the glowing circle task, participants are presented with a sequence of glowing circles that they must reproduce in the same order.

**FIGURE 1:**
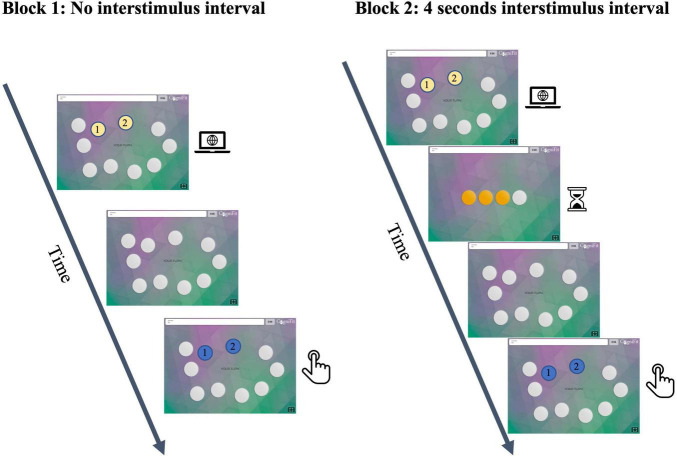
Visuospatial working memory task. The participants were given instructions to press circles in a specific order under two different conditions: Block 1, where there was no break between trials, and Block 2, where a 4-s break was introduced between each trial.

In this study, participants were exposed to 10 circles on the screen in a determined set of coordinates. The computer flashed some of these circles for 2 s on each trial that participants had to click in the same order. The task was presented in two blocks of different interstimulus intervals. The first block had no interstimulus interval, while the second block had an interstimulus interval of 4 s. Each block had nine trials. The first block started with two flashing circles, while the second block started with the maximum correct trials from the first block minus 3. Each subsequent trial in a block added an extra flashing circle. Figure 1 contains a visual representation of the task. If a user responded incorrectly to one trial of a given difficulty level, another trial of the same difficulty level would be presented. The task would stop upon incorrect completion of two trials of the same difficulty level. Data are presented in z-scores, obtained by standardizing accuracy rates by using the following formula:


$$z=\frac{x-\text{μ}}{\text{σ}}$$

where: z is the z-score; *x* is the raw accuracy data; μ is the mean (average) of the distribution; σ is the standard deviation of the distribution. The z-scores were calculated using reference databases with a sample size of 915,400 users.

#### 2.2.2. Phonological working memory

Participants completed the CogniFit game “The Numbers” to measure this ability. This task is a variation of the Wechsler Digit Span test, which measures phonological short-term and working memory (Wechsler, 1981). In this task, the participant is presented with a progressively longer series of numbers that must be recalled in the same order they were presented.

**FIGURE 2:**
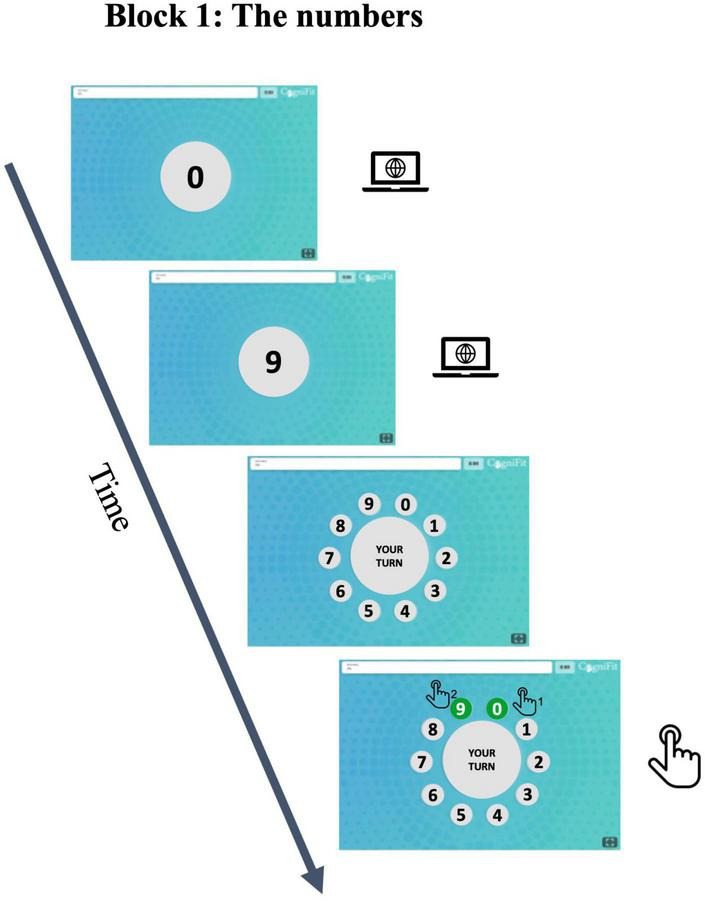
Phonological working memory. The participants were given instructions to memorize a sequence of numbers and subsequently reproduce them in the exact order in which they were presented.

In this study, participants were presented with a large circle in the middle of the screen displaying a random set of numbers sequentially for 1 s, followed by a 1-s delay. Once the set of numbers was presented, ten smaller circles appeared around the central circle, each containing a number from 0 to 9 arranged clockwise. Participants were expected to click on the numbered circles in the correct order to reproduce the series that was shown previously. After each successful trial, a number was added to the series of numbers. The block had nine trials. As in the case of the preceding task, here, participants also had two attempts to complete a trial of a given difficulty level. The task was discontinued after two consecutive errors in trials of the same difficulty level. Figure 2 contains a visual representation of the task. Data are presented in z-scores, obtained by standardizing accuracy rates. The z-scores were calculated using reference databases with a sample size of 883,192 users.


$$z=\frac{x-\text{μ}}{\text{σ}}$$

#### 2.2.3. Sociodemographic and wellbeing questionnaire

The wellbeing questionnaire asks participants about their physical, psychological, and social wellbeing. The questionnaire also asks about more general issues outside these themes, such as manual dominance. Given that exercise and sleep quality are well-known modulators in the relationship between age and cognitive functioning, we included the questions that explored them in our analysis. In this sense, we included the yes/no questions (1) Do you get 7–9 h of sleep each night? and (2) Do you exercise often. In terms of potential cognitive reserve factors, considerations included whether the participant is currently engaged in a profession (Are you presently employed?) and whether they are proficient in multiple languages [Do you speak two (or more) languages fluently?].

### 2.3. Data analysis plan

To conduct our analyses, we used the software RStudio, version 4.2.1 (R Core Team, 2021). To fulfill the purpose of the study, which was to explore the relationship between working memory and age, we conducted a linear regression for each dependent variable (visuospatial and phonological working memory test scores). The independent variables were age but also included physical activity and sleep duration as control variables and an age × gender interaction.

Concerning cognitive reserve factors, supplementary analyses of covariance (ANCOVA) were conducted with gender and cognitive reserve variables as fixed effects and age as a covariate. To determine whether these factors nullified the main effects examined in this study. *Post hoc* analyses were conducted using the Bonferroni multiple comparisons test in cases where significant interactions were found.

## 3. Results

Before individually assessing each task, we ran a Pearson’s correlation analysis to confirm that the participants’ scores in the phonological and visuospatial working memory tasks were significantly correlated (*r* = 0.36, *p* < 0.001), as expected for two tasks that measure partially overlapping cognitive skills.

**FIGURE 3:**
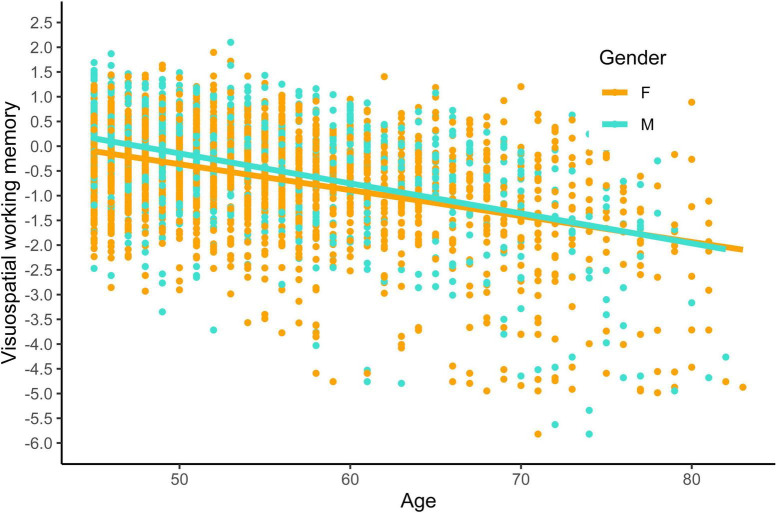
Visuospatial working by gender and age. Visuospatial working memory was calculated using z-scores, while age was measured in years. Participants’ gender was indicated by F for female and M for male.

First, we examined the relationship between visuospatial working memory, age, gender, sleep duration, physical activity, and an interaction effect between age and gender. Overall, the model accounted for a moderate amount of variance in visuospatial working memory [*R*^2^ = 0.233, *F*(5, 3,206) = 195.2, *p* < 0.001]. Our results showed that age significantly predicts visuospatial working memory [β(unstandardized) = −0.066, *t*(3,206) = −23.874, *p* < 0.001], with older age-associated with worse performance. Additionally, gender significantly predicted visuospatial working memory [β(unstandardized) = 0.959, *t*(3,206) = 3.495, *p* < 0.001], in which male participants obtained better scores than female participants. The age × gender interaction was significant [β(unstandardized) = −0.015, *t*(3,206) = −2.955, *p* = 0.003], indicating that the difference between genders was more marked at younger ages, with older participants showing negligible differences depending on their gender. See Figure 3 for a visual representation.

The supplementary ANCOVA for visuospatial working memory showed a main effect of being professionally active [*F*(1, 3,245) = 40.466; *p* < 0.001; η^2^ = 0.010], as well as an interaction between employment*gender [*F*(1, 3,245) = 7.018; *p* = 0.008; η^2^ = 0.002]. Regarding this interaction, significant differences were observed in professionally active women ($\bar{x}$ = −0.439) compared with those who are not ($\bar{x}$ = −0.932; *t* = −3.523; *p* = 0.003). Similarly, a significant difference in scores was observed between professionally active men ($\bar{x}$ = −0.255) and those who are not ($\bar{x}$ = −0.991; *t* = −5.442; *p* < 0.001). Significant differences were also observed in comparing non-professionally active women and professionally active men (MD = −0.408; *t* = −7.202; *p* < 0.001). However, no differences were observed between non-professionally active men and professionally active women (MD = −0.149; *t* = −2.299; *p* = 0.129). When women and men were professionally active, women displayed significantly lower scores than men (MD = −0.241; *t* = −4.387; *p* < 0.001). In the case where neither continued with employment, no significant differences were observed ($\bar{x}$ = −0.018; *t* = −0.287; *p* = 1).

**FIGURE 4:**
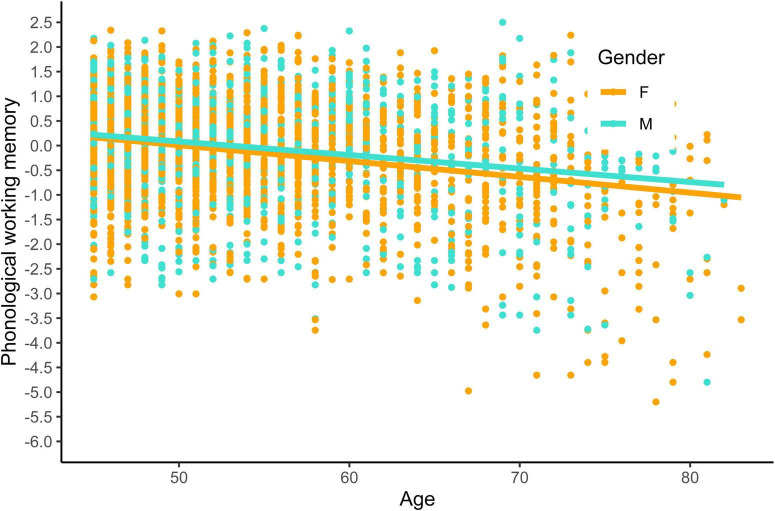
Phonological working memory by gender and age. Phonological working memory was calculated using z-scores, while age was measured in years. Participants’ gender was indicated by F for female and M for male.

Second, we examined the relationship between phonological working memory, age, gender, sleep duration, and physical activity. In addition, the model tested for an interaction effect between age and gender. Overall, the model accounted for a small amount of variance in phonological working memory [*R*^2^ = 0.056, *F*(5, 3,206) = 37.79, *p* < 0.001]. Our results showed a significant negative effect of age on phonological working memory test scores [β(unstandardized) = −0.031, *t*(3,206) = −11.661, *p* < 0.001]. Gender, sleep quality, and physical activity did not significantly predict phonological working memory. The interaction between age and gender was also not significant. See Figure 4 for a visual representation.

The supplementary ANCOVA for phonological working memory showed a main effect of being professionally active [*F*(1, 3,245) = 30.922; *p* < 0.001; η^2^ = 0.009], as well as a main effect of fluency in other languages [*F*(1, 3,245) = 8.612; *p* = 0.003; η^2^ = 0.002]. The analysis also showed an interaction between gender*employment [*F*(1, 3,245) = 7.762; *p* = 0.005; η^2^ = 0.002]. Regarding this interaction, professionally active women ($\bar{x}$ = −0.023) significantly outperformed non-professionally active women ($\bar{x}$ = −0.274; *t* = −5.493; *p* < 0.001). Likewise, a significant difference in scores was observed between professionally active men ($\bar{x}$ −0.137) and non-professionally active men ($\bar{x}$ = −0.349; *t* = −6.794; *p* < 0.001). Significant differences were also observed when comparing non-professionally active women and professionally active men (MD = −0.305; *t* = −5.557; *p* < 0.001). Similarly, differences were observed between non-professionally active men and professionally active women (MD = −0.166; *t* = −2.658; *p* = 0.047). When men and women were professionally active, women displayed significantly lower scores than men (MD = −0.213; *t* = −4.313; *p* < 0.001). In the case where neither continued with professional activity, no significant differences were observed (MD = −0.033; *t* = −0.463; *p* = 1).

## 4. Discussion

This study aimed to determine if user-generated data on cognitive abilities was sensitive to the well-known effects of aging on memory. The results showed a progressive loss of phonological and visuospatial working memory capacity with increased age, confirming earlier reports in the literature (Bopp and Verhaeghen, 2005, 2020; Pliatsikas et al., 2019).

We also found a gender effect showing that male participants significantly outperformed female participants in visuospatial working memory tasks but not phonological working memory, confirming the results of previous studies (Fournet et al., 2012; Morais et al., 2018). A possible explanation for gender differences in visuospatial working memory is the reported visuospatial advantage for male individuals (Voyer et al., 2017). This result, however, contradicts (Choi et al., 2014), who found gender differences in phonological working memory in elderly individuals, in which male participants outperformed female participants. However, although significant, the explained variance of gender was negligible.

This result, however, contradicts who found gender differences in phonological working memory in elderly individuals, in which male participants outperformed female participants. However, although significant, the explained variance of gender was negligible.

In addition to finding a gender effect, we also found an age × gender interaction for the visuospatial working memory tasks. Male participants show a steeper decline than female participants. There is conflicting evidence about this age × gender interaction, as there are studies that confirm our results (Lipnicki et al., 2013; McCarrey et al., 2016; Pliatsikas et al., 2019) while other studies show that women tend to have a faster cognitive decline than men (Zhang, 2006; Lin et al., 2015; Levine et al., 2021). The literature suggests that the effects of age-related neuroendocrine changes on cognitive performance should be studied separately by sex (Hampson, 1990), given the relevant impact of testosterone levels on memory, executive functions, and spatial task execution (Holland et al., 2011), as well as the influence of estrogens on verbal fluency, spatial tasks, and memory in women (Hampson, 1990). Hormonal levels not only change across the lifespan but also exhibit cyclical patterns.

We included sleep quality and exercise in the model since these variables have been shown to affect cognitive performance (Angevaren et al., 2010; Lee et al., 2015; Brocklebank et al., 2021; Joo et al., 2021; Song and Park, 2022). However, we did not find an effect of the variables when considered. This does not imply that sleep or exercise do not contribute to cognitive health but that the instruments used to measure them might not have been sensitive enough to capture the effect. They were measured with a single yes/no question that does not fully describe the nature of these lifestyle choices. Further data collection could contemplate using a validated psychometric instrument to better explore the nuances of sleep quality and physical exercise to evaluate their effect on older individuals’ cognitive functioning. In addition, online computerized cognitive assessment platforms could also integrate physiological data obtained by wearable devices that continuously track sleep and physical activity data. More accurate and dynamic data will improve any cognitive evaluation platform’s predictive power.

Similarly, additional analyses were conducted exploring the interaction of factors considered relevant to participants’ cognitive reserve. In this regard, we observed that the effects of participants’ age and gender persisted on their scores, both in visuospatial and phonological working memory. Furthermore, we noted a protective effect of participants’ professional activity on their scores in both explored domains of working memory. This supports the notion that continuous occupational engagement helps maintain cognitive function (Adam et al., 2013; Alvares Pereira et al., 2022). Likewise, we observed an effect of fluency in other languages on phonological working memory. In this regard, the evidence suggests that bilingualism contributes to preserving cognitive function (Bialystok et al., 2007; Guzmán-Vélez and Tranel, 2015; Antón et al., 2019; Bialystok, 2021).

The present study has several limitations that should be considered before its interpretation. The first limitation is that the evaluations were conducted in an uncontrolled and naturalistic environment, so researchers did not have control over the conditions in which these data were gathered. The second limitation is that the data presented here comes from a cross-sectional design, which does not account for an actual cognitive decline, but describes lower performance with increasing age. Further studies should consider collecting multiple data points of users to characterize cognitive function change across time and evaluate the effect of exposure to the tests (Salthouse, 2019). This is especially important when discriminating between age-associated cognitive decline and dementia, given that one of their differentiating factors is the rate at which deterioration occurs (Deary et al., 2009). Also, future investigations should delve more extensively into the relationship between occupational activity, bilingualism, and visuospatial and phonological working memory, especially considering the interactions highlighted in the supplementary analyses of this study.

Notwithstanding these limitations, this study is relevant given the large sample tested and the need for developing robust platforms that continuously monitor aging individuals’ cognitive function and lifestyle characteristics. Such platforms have the potential to characterize better dynamic cognitive functions as well as the potential buffering mechanisms to prevent pathological cognitive decline by providing individualized, targeted strategies to approach age-associated cognitive decline. More research needs to be conducted to better characterize individual variability and factors that predict cognitive decline in the aging population (Erickson et al., 2022). If used as a recurrent evaluation tool, technological tools, such as app-based screeners, could better characterize these risk factors and eventually identify individuals at risk for developing pathological cognitive decline. Such an approach opens doors to a new potential for intervention and the use of artificial intelligence trained to recognize patterns in large amounts of data. Artificial intelligence could aid in creating individualized intervention plans well-suited to contexts, risk factors, lifestyles, and needs, contributing to the wellbeing of the aging population.

## Data availability statement

The original contributions presented in this study are included in the article/supplementary material, further inquiries can be directed to the corresponding author.

## Ethics statement

The studies involving humans were approved by the Ethics Board of Universidad Nebrija (UNNE-2020-008). The studies were conducted in accordance with the local legislation and institutional requirements. The participants provided their written informed consent to participate in this study.

## Author contributions

LS-V contributed substantially to the conception of the work, interpretation of the data, and drafted the manuscript. DC-B and HM-S contributed substantially to the conception of the work and analyzed and interpreted the data. JD contributed substantially to the conception of the work and revised the manuscript critically for important intellectual content. All authors provided approval for publication of the content and agreed to be accountable for all aspects of the work in ensuring that questions related to the accuracy or integrity of any part of the work are appropriately investigated and resolved.
